# Comparing estimates of influenza-associated hospitalization and death among adults with congestive heart failure based on how influenza season is defined

**DOI:** 10.1186/1471-2458-8-59

**Published:** 2008-02-13

**Authors:** Carolyn Sandoval, Stephen D Walter, Paul Krueger, Mark B Loeb

**Affiliations:** 1 Department of Clinical Epidemiology and Biostatistics, McMaster University, Hamilton, Ontario, Canada

## Abstract

**Background:**

There is little consensus about how the influenza season should be defined in studies that assess influenza-attributable risk. The objective of this study was to compare estimates of influenza-associated risk in a defined clinical population using four different methods of defining the influenza season.

**Methods:**

Using the Studies of Left Ventricular Dysfunction (SOLVD) clinical database and national influenza surveillance data from 1986–87 to 1990–91, four definitions were used to assess influenza-associated risk: (a) three-week moving average of positive influenza isolates is at least 5%, (b) three-week moving average of positive influenza isolates is at least 10%, (c) first and last positive influenza isolate are identified, and (d) 5% of total number of positive isolates for the season are obtained. The clinical data were from adults aged 21 to 80 with physician-diagnosed congestive heart failure. All-cause hospitalization and all-cause mortality during the influenza seasons and non-influenza seasons were compared using four definitions of the influenza season. Incidence analyses and Cox regression were used to assess the effect of exposure to influenza season on all-cause hospitalization and death using all four definitions.

**Results:**

There was a higher risk of hospitalization associated with the influenza season, regardless of how the start and stop of the influenza season was defined. The adjusted risk of hospitalization was 8 to 10 percent higher during the influenza season compared to the non-influenza season when the different definitions were used. However, exposure to influenza was not consistently associated with higher risk of death when all definitions were used. When the 5% moving average and first/last positive isolate definitions were used, exposure to influenza was associated with a higher risk of death compared to non-exposure in this clinical population (adjusted hazard ratios [HR], 1.16; 95% confidence interval [CI], 1.04 to 1.29 and adjusted HR, 1.19; 95% CI, 1.06 to 1.33, respectively).

**Conclusion:**

Estimates of influenza-attributable risk may vary depending on how influenza season is defined and the outcome being assessed.

## Background

Complications associated with influenza infection have been well described [1-5]. While the influenza season usually begins in November and ends in April in North America, the exact start and stop dates are not fixed. Since periods of circulating influenza vary on both an annual and regional basis, careful definitions of exposure are needed when assessing the impact on outcomes such as hospitalization and death. The Centers for Disease Control and Prevention (CDC) defines the start and stop of the influenza season using a 5% moving average of positive influenza isolates obtained from weekly national surveillance testing. However, many other definitions are also used, including 5% of the total number of positive isolates obtained for the whole season [6,7], the first and last positive isolate identified [8-12], the "winter season" [13,14], and when positive isolates exceed 10% of total tested [4,15]. There is little consensus about exactly how the influenza season should be defined in studies assessing influenza-attributable risk.

To our knowledge, there have been no published studies comparing the effect of using different differences on estimates of complications of influenza. We previously conducted an analysis to assess the effect of circulating influenza season on hospitalization and death among patients with congestive heart failure (CHF) [16]. We report in this paper a comparison of four different methods to define the influenza season and assessed the effect of these estimates on influenza-attributable hospitalization and mortality using a database of patients with CHF.

## Methods

To assess influenza-associated risks of hospitalization and death (separately), we used the database of the Studies of Left Ventricular Dysfunction (SOLVD) trials, which included patients with physician-diagnosed CHF that were followed prospectively for 5 years from 1986–1991 [17]. Data collected for the Studies of Left Ventricular Dysfunction (SOLVD) trials were used as the clinical database. SOLVD consisted of two double-blind, placebo-controlled randomized trials that examined the effect of the angiotensin-converting-enzyme inhibitor enalapril on morbidity and mortality in moderately severe CHF patients (12, 13). Participants with CHF and left ventricular ejection fractions ≤ 35% who were already taking drugs other than an angiotensin-converting-enzyme inhibitor were eligible. Participants were ineligible if they were over the age of 80 years or if they had any of the following: hemodynamically serious valvular disease requiring surgery, unstable angina, angina thought to be serious enough to require revascularization procedures, myocardial infarction in the previous month, severe pulmonary disease, serum creatinine higher than 177 umol per litre, or any other disease that might substantially shorten survival or impede participation in a long-term trial. Asymptomatic CHF patients, defined as those with no clinical symptoms of CHF, were enrolled in the SOLVD Prevention trial, while symptomatic CHF patients were enrolled in the SOLVD Treatment trial. Participants were followed prospectively from 1986 to 1991. During this period, 39,924 patients with ejection fractions ≤ 35% were identified. Of these 6.4% or 2,569 were enrolled in the treatment trial and 7.4% or 4,228 were enrolled in the prevention trial. The reasons for exclusion included the following: use of an angiotensin-converting-enzyme inhibitor (28%), cardiovascular problems (12%), contraindications to use of an angiotensin-converting-enzyme inhibitor (11%), lack of consent (11%), administrative reasons (21%), cancer or other life-threatening disease (12%), other reasons (5%). There were 24 study sites of which 21 were located throughout the continental US, two in Canada, and one in Belgium (12, 13). Each was comprised of one to eight hospitals. Only participants from the 21 US sites were included in our analysis, since weekly influenza isolate data were available. A small number (<1%) of the participants were excluded because the exact date of their first hospitalization post-randomization could not be determined, leaving 5,448 people in the study population.

National influenza surveillance data from 1986–1987 to 1990–1991 were obtained from the Influenza Branch of the Centers for Disease Control and Prevention. The weekly number of isolates submitted for testing each year and the weekly number of positive tests obtained each year (by virus type and subtype) were used identify the start and stop of the influenza season using four methods. Isolate data and SOLVD study sites were classified into four regions: Central, Northeast, West, and South.

The following four methods were used to define the start and stop of the influenza seasons: 5% moving average, 10% moving average, first and last isolate, and 5% total season isolates. The *5% moving average *refers to the first and last week during which the three-week moving average of influenza isolates submitted for testing was at least 5 percent positive. The *10% moving average *refers to the first and last week in which the three-week moving average of influenza isolates submitted for testing was at least 10 percent positive. The *first/last isolate *refers to the weeks in which the first and last positive influenza isolate for that season was identified. The *5% total season *refers to the weeks during which the number of positive influenza isolates obtained was at least 5% of the total number of positive influenza isolates obtained for the entire season.

Hospitalization and mortality rates during the influenza seasons and non-influenza seasons were compared for each different definition of the influenza season. All patient-days at risk during the influenza season and non-influenza seasons were calculated based on date of randomization, dates of the beginning and end of each influenza season, and the date of hospitalization, death or last SOLVD visit. Incidence rates of first-time hospitalization were determined by dividing the number of events during a given time period (influenza or non-influenza season) by the total days at risk for eligible individuals during the same period. Days at risk were calculated from the date of enrolment into the study until the date of the first hospitalization or, if no hospitalization occurred, until the date of the final follow up visit. We calculated the rate ratio (relative risk) and 95% confidence intervals (CI) for hospitalization and mortality during the combined influenza seasons when compared to combined non-influenza seasons.

Cox regression was used to assess the effect of exposure to flu season on all-cause hospitalization and death for each different definition of the influenza season and adjusted hazard ratios were compared. Adjustment for sex, age, enalapril use, severity of congestive heart failure (based on the New York Heart Classification) [18] and co-morbidities (using the Charlson Index) [19] was performed. The influenza season for each participant was defined as a time-dependent variable based on his/her randomization date and follow-up time, and on the dates of the influenza season of his/her region during each study year. In our previous analysis, daily temperature was forced into the multivariable model [16]. However, since this variable was not significant, we did not include it in the present set of analyses.

To compare the effects of the hazard ratio and time of exposure to influenza season of the difference definitions of the influenza season, we calculated the proportion of hospitalizations and deaths attributable to influenza (hazard ratio – 1/hazard ratio) and multiplied this by the number of events (hospitalizations or deaths) within the influenza season however defined. Using patient-days as the denominator and the attributable events as the numerator, we calculated the annualized rate of attributable hospitalizations and deaths.

## Results

### Risk of Hospitalization during the Influenza Season

Hospitalization rates during the influenza and non-influenza season for all-years combined when the four methods were used are shown in Table 1. The number of hospitalizations was highest when the influenza season was represented by a larger period of calendar time. When more restricted definitions were used, fewer hospitalizations were categorized as influenza-related events.

**Table 1:** Comparing hospitalization rates during the influenza season and non-influenza season using different methods of defining the influenza season, 1986–87 to 1990–91.

	Influenza Season*	Non-Influenza Season*	Relative Risk (95% Confidence Interval)
5% Moving Average			
Rate	11.39	10.52	1.08 (1.01–1.16)
Events, No.	1204	2177	
Patient-days, No.	1057293	2069683	
10% Moving Average			
Rate	11.34	10.63	1.07 (0.99–1.15)
Events, No.	901	2480	
Patient-days, No.	794239	2332737	
First/Last			
Rate	11.26	10.35	1.09 (1.01–1.16)
Events, No.	1777	1604	
Patient-days, No.	1577740	1549236	
5% Total Season			
Rate	11.18	10.72	1.04 (0.96–1.13)
Events, No.	687	2694	
Patient-days, No.	614407	2512569	

* Rates are per 10,000 patient-days

The calculated relative risk (unadjusted) showed that overall hospitalization rates during the influenza season were higher than hospitalization rates during the non-influenza season for all methods (5% moving average: RR, 1.08; 95% CI, 1.01 to 1.16; 10% moving average: RR, 1.07; 95% CI, 0.99 to 1.15; first/last: RR, 1.09; 95% CI, 1.01 to 1.13; 5% total: RR, 1.04; 95% CI, 0.93 to 1.13). However, only the 5% moving average and first/last isolate definitions showed a significant difference between rates during the influenza season and non-influenza season. Hospitalization rates during the influenza season were relatively similar when different definitions were used. The highest and lowest hospitalization rates during the influenza season were obtained when the 5% moving average and 5% total season definitions were used, respectively.

The unadjusted hazard ratio associated with exposure to influenza was determined by constructing Cox models with only the influenza season variable (Table 2). For the hospitalization model, the unadjusted hazard ratios associated with influenza using 5% moving average, 10% moving average, first/last isolate and 5% total season isolates were 1.09 (95% CI, 1.02 to 1.17), 1.04 (95% CI, 0.97 to 1.11), 1.07 (95% CI, 0.99 to 1.14), and 1.07 (95% CI, 0.99 to 1.15), respectively (Table 2). The hazard ratios for hospitalization associated with influenza remained relatively constant when adjusted for other variables for all definitions except for the 10% moving average, which increased by five percent (Table 2). However, when the hazard ratio was adjusted for other variables using the 10% moving average, influenza season was found to be significant (as it was for other definitions). The rate of attributable excess hospitalizations was variable depending on the definition use. Notably, the annualized rate of hospitalizations was highly variable, ranging from 5.95 (5% total season) to 15.41 (first/last) hospitalizations per 1,000 patient-years depending on the definition used.

**Table 2:** Comparison of estimated risk of hospitalization associated with influenza season using different methods of defining the influenza season.

Flu Season Definition	Unadjusted Hazard Ratio (95% CI)	Adjusted Hazard Ratio* (95% CI)	Number of hospitalizations attributable to influenza	Rate of Attributable Excess hospitalizations** (95%CI)
5% Moving Average	1.09 (1.02–1.17)	1.10 (1.04–1.18)	109	12.72 (10.35 to 15.47)
10% Moving Average	1.04 (0.97–1.11)	1.09 (1.02–1.17)	74	8.64 (6.74 to 10.58)
First/Last Isolate	1.07 (0.995–1.14)	1.08 (1.01–1.16)	132	15.41 (12.54 to 18.74)
5% Total Season Isolates	1.07 (0.989–1.15)	1.08 (1.00–1.17)	51	5.95 (4.43 to 7.82)

CI = confidence interval;

* adjusted for trial, age group, NYHA class, co-morbidity index score, enalapril therapy, ejection fraction, and study site.

**Rates are per 1,000 patient-years

### Risk of Death during the Influenza Season

Death rates for all years combined during influenza and non-influenza seasons are shown in Table 3. Using the four definitions, relative risk of death were as follows: 5% moving average: RR, 1.09; 95% CI, 0.97 to 1.21; 10% moving average: RR, 1.08; 95% CI, 0.95 to 1.21; first/last: RR, 1.15; 95% CI, 1.02 to 1.27; 5% total: RR, 1.06; 95% CI, 0.92 to 1.20. The highest and lowest death rates during the influenza season were obtained when the first/last isolate and 5% moving average definitions were used, respectively.

**Table 3:** Comparing death rates during the influenza season and non-influenza season using different methods of defining the influenza season, 1986–87 to 1990–91.

	Influenza Season*	Non-Influenza Season*	Relative Risk (95% Confidence Interval)
5% Moving Average			
Rate	27.10	24.87	1.09 (0.97–1.21)
Events, No.	475	841	
Patient-days, No.	1752471	3382155	
10% Moving Average			
Rate	27.13	25.11	1.08 (0.95–1.21)
Events, No.	358	958	
Patient-days, No.	1319485	3815141	
First/Last			
Rate	27.34	23.86	1.15 (1.02–1.27)
Events, No.	713	603	
Patient-days, No.	2607851	2526805	
5% Total Season			
Rate	26.79	25.35	1.06 (0.92–1.20)
Events, No.	271	1045	
Patient-days, No.	1011662	4122964	

* Rates are per 100,000 patient-days

For the death model, the unadjusted hazard ratios associated with the influenza season using 5% moving average, 10% moving average, first/last isolate and 5% total season isolates were: 1.09 (95% CI, 0.97 to 1.21), 1.08 (95% CI, 0.95 to 1.21), 1.15 (95% CI, 1.02 to 1.27), and 1.06 (95% CI, 0.92 to 1.20), respectively (Table 4). The unadjusted hazard ratios were similar to the unadjusted relative risk obtained in the incidence rate analysis. Depending on which definition was used, the hazard ratios for death associated with the influenza season increased by five to eight percent when adjusted for other variables (Table 4). As seen with hospitalization, the rate of attributable excess deaths was variable depending on the definition use. There was striking variation in the annualized rate of deaths attributable to influenza, ranging from 0.71 (5% total season) to 8.10 (first/last isolate) deaths per 1,000 patients per year depending on the definition used.

**Table 4:** Comparison of estimated risk of death associated with influenza season using different methods of defining the influenza season.

Flu Season Definition	Unadjusted Hazard Ratio (95% CI)	Adjusted Hazard Ratio* (95% CI)	Number of deaths attributable to influenza	Rate of Attributable Excess Deaths** (95%CI)
5% Moving Average	1.10 (0.98–1.22)	1.16 (1.04–1.29)	66	4.69 (3.61 to 5.96)
10% Moving Average	1.03 (0.92–1.15)	1.08 (0.97–1.21)	27	1.92 (1.27 to 2.79)
First/Last Isolate	1.11 (0.995–1.24)	1.19 (1.06–1.33)	114	8.10(6.59 to 9.85)
5% Total Season Isolates	0.997 (0.881–1.13)	1.04 (0.92–1.18)	10	0.71 (0.34 to 1.31)

CI = confidence interval;

* adjusted for trial, age group, NYHA class, co-morbidity index score, enalapril therapy, ejection fraction, and study site.

**Rates are per 1,000 patient-years

## Discussion

Overall, the hospitalization models were very similar and the covariates had similar direction and size of effect. Most notably, exposure to influenza was statistically significant in all adjusted models, suggesting that circulating influenza had an effect on hospitalization during the entire circulating period as well as during the peak circulating periods. The overall risk of hospitalization during the influenza season varied between 8–10% for the four methods. In all adjusted models, irrespective of which influenza definition was used, the direction and magnitude of effects for the individual-level covariates (being symptomatic for CHF, being older, not being on enalapril, having a higher Charlson score, having a higher NYHA class and having a lower ejection fraction) also remained constant (data not shown). Estimated risk of hospitalization during the influenza season dropped by one or two percent when definitions other than the standard CDC definition were used (i.e. 5% moving average) (Table 2).

Only small differences between unadjusted relative risk and unadjusted hazard ratios occurred when the various definitions to determine influenza-associated risk were compared. The relative risk comparing the overall event rates during the influenza season with the non-influenza season were cruder estimates of influenza – associated risk when compared to the hazard ratio estimates, which calculated the absolute baseline hazard for hospitalization over time. The unadjusted and adjusted hazard ratios were similar. The effect size of influenza season remained constant despite the higher effect sizes of the individual-level covariates.

For the death model, the adjusted hazard ratios varied considerably between the four methods (Table 4). Unexpectedly, the model using a 10% moving average did not show the greatest effect on death. This would have been expected since it represented the strictest definition of the influenza season. However, influenza season showed a significant effect on death only when the 5% moving average and first/last isolate definitions were used. Additionally, the greatest effect size (adjusted HR, 1.19) was seen when the first/last definition was used. One possible explanation for this is that by increasing the length of the influenza season, the 5% moving average and the first/last isolate definitions limited the reference period to summer months when there is less likely to have circulating respiratory viruses.

Only the adjusted multivariable models using the 5% moving average and first/last isolate definitions showed an association between circulating influenza and hospitalizations as well as an association between influenza and death. The first/last isolate definition was the broadest definition. The annualized influenza attributable rates of hospitalization and death were quite variable depending on the definition used (Tables 2 and 4). It could be that while all influenza-attributable events were identified, hospitalizations and deaths not related to influenza (but due to co-circulating viruses such as respiratory syncitial virus) may have been incorrectly attributed to influenza. The 5% total season measure will likely identify most influenza attributable cases, but since the number of tests done each week and each season were not considered, this method may not be reliable during seasons in which influenza testing is low. The fact that the estimates of risk were consistent when using hospitalization but not mortality raises the importance of the effect of the outcome.

Our analysis has several limitations. Influenza vaccination has been shown to be effective at reducing morbidity and mortality associated with influenza infection [19]. No reliable information was available for each study participant on influenza vaccination. However, in 1998, only 43% of people with chronic pulmonary and cardiac conditions were vaccinated against influenza and it is likely that vaccination coverage was even lower during the study period used here [20]. Thus, vaccination status probably did not affect the reported rates and the proposed models here.

As well, there was no individual-level data on exposure to and infection with influenza. Our assumption is that study patients living in a particular geographic region were exposed to the virus during the circulating period and that this exposure increased the probability of hospitalization or death.

Influenza A(H3N2) is associated with more serious morbidity and mortality than influenza A(H1N1) and influenza B [21]. In our analysis, the cumulative risk was determined by assuming that the risks of hospitalization and death was similar across years, but the predominant influenza type differed by study year. During our study period, years 2 and 4 were predominantly A(H3N2), year 1 was mostly A(H1N1) and years 3 and 5 were mostly influenza B (Table 5). Furthermore, the predominant influenza subtype may not be the same in all regions, thus potentially affecting risk geographically. In our case, however, the predominant type was the same in all regions for most years.

**Table 5:** Start and stop weeks of influenza season using various definitions from 1986–87 to 1990–91 influenza seasons by United States regions. Patients in the study were enrolled from a study site within one of the four regions.

**Region**	**Year**	**First last isolate**		**10% moving average**		**5% moving average**		**5% all isolates**	
		Start	Stop	Start	Stop	Start	Stop	Start	Stop

Central	86–87	49	17	51	5	50	9	51	7
Northeast		44	11	50	5	49	6	50	4
South		45	16	53	9	51	10	1	6
West		45	8	51	5	48	6	52	5
									
Central	87–88	46	22	4	18	3	21	4	13
Northeast		1	22	7	14	6	23	8	13
South		50	25	4	14	3	14	4	13
West		52	20	2	3	1	9	2	11
									
Central	88–89	47	19	2	12	1	15	3	10
Northeast		47	18	2	18	1	18	4	10
South		50	19	51	15	51	20	3	11
West		51	17	8	10	4	13	3	13
									
Central	89–90	47	17	51	11	50	12	52	9
Northeast		48	21	1	11	52	12	2	10
South		48	12	50	12	50	13	51	8
West		46	11	3	6	52	9	51	8
									
Central	90–91	48	19	5	14	3	14	4	13
Northeast		45	22	3	10	50	17	3	8
South		40	19	1	16	51	19	3	8
West		52	21			5	9	5	12

Attributing a particular influenza type to each participant was not possible since there was no individual level data available that could identify the exact influenza subtype that each person was exposed to and for how long.

It must also be acknowledged that hospitalizations for heart-related causes are higher during winter months compared to summer months [22-26]. The winter season and the influenza season usually fall during similar calendar periods, suggesting that there may be confounding due to more hospitalizations occurring during the winter. However, since it was not controlled for in these population-based studies, there was no way to determine whether the effect reported was due to respiratory viruses versus other reasons, such as lower immune response during colder temperatures, less frequent physical activity, and/or depression.

## Conclusion

How influenza season is defined can have an important influence on estimates of association when evaluating influenza-attributable risk. The definitions can have a differential impact depending on the outcome being considered.

## Competing interests

The author(s) declare that they have no competing interests.

## Authors' contributions

ML, PK, SDW are responsible for the conception and design of the study. CS is mainly responsible for the analysis with input from SDW, ML, and PK. All authors were involved in drafting the manuscript and revising it. All authors have given final approval.

## Pre-publication history

The pre-publication history for this paper can be accessed here:


